# Optical Caliper for Contactless Measurement of Plant Stem Diameter

**DOI:** 10.3390/s26062007

**Published:** 2026-03-23

**Authors:** Naomi van der Kolk, Daan Boesten, Willem van Valenberg, Steven van den Berg

**Affiliations:** Photonics Research Group, The Hague University of Applied Sciences, Rotterdamseweg 137, 2628 AL Delft, The Netherlands

**Keywords:** optical caliper, stem diameter, head thickness, contactless measurement, non-invasive monitoring, precision agriculture, plant phenotyping, greenhouse monitoring, shadow formation, image processing

## Abstract

Precision greenhouse agriculture enhances plant health and crop yields by continuously monitoring key plant parameters. Stem diameter is such a parameter and is monitored to support decisions on plant care. However, traditional contact-based methods induce thigmomorphogenic effects that impact plant growth. Here, we introduce the Optical Caliper (OC), a novel contactless device for precise, non-invasive stem diameter measurement. The OC operates by projecting a collimated light beam to cast a shadow of the stem onto a high-resolution image sensor. The shadow size is a measure for the stem diameter. Controlled laboratory tests show the OC offers an accuracy comparable to that of a Digital Caliper (DC). Field trials on irregular tomato and cucumber stems demonstrate a repeatability of 0.1–0.2 mm. The OC’s non-invasive design and high repeatability exceed the performance of a DC, making it particularly suited for accurately monitoring soft, variable plant structures. Bringing the advantage of avoiding thigmomophogenic effects and thus optimizing crop yield, the OC is a promising tool for high-throughput plant phenotyping and precision agriculture applications.

## 1. Introduction

Precision greenhouse agriculture attempts to improve plant health and increase crop yields by tracking plant characteristics such as stem diameter [1] and leaf area index [2]. The level of control a grower has on the crop production process partially depends on the precision with which these characteristics are measured, as these features are directly related to the growth of the plant. Based on measurements of the climate of the greenhouse and the state of the crops, growers make decisions for yield optimization.

In greenhouse tomato cultivation, assessing plant health involves tracking structural and physiological traits. The stem diameter of plants provides valuable insight into the plant’s water and nutrient status, photosynthetic activity, and potential stress levels [3]. Stem diameter variations over time can be measured using dendrometers or linear variable displacement transducers, as these instruments can measure small variations in the order of several micrometers [4,5]. In certain crops, such as tomatoes, head thickness—defined as the stem diameter measured approximately 25 cm below the growing apex—is routinely monitored as a key phenotypic trait. This parameter is conventionally assessed using a digital caliper (DC). Although DCs provide sufficient accuracy for quantifying apex head thickness, their primary limitation is the requirement for direct physical contact with the plant, which can potentially damage sensitive apical tissues or disrupt ongoing growth processes. Many plants are sensitive to being touched and, in response, can severely reduce their growth rate, an effect known as thigmomorphogenesis [6,7]. For this reason, growers want to be able to limit contact with plants to a minimum.

Although several non-contact methods have been proposed for monitoring stem diameters, none have achieved sufficient accuracy to replace digital caliper measurements with resolutions with 0.1 mm or better [8]. Camera-based approaches using edge detection and reference scaling or depth sensing for pixel to dimension conversions are most common. Nock et al. (2013) reported errors of ±2 mm for stems above 7 mm and could not measure stems below 6.5 mm with a camera and structured-light system [9]. Batz et al. (2016) achieved a Root Mean Square Error (RMSE) of 3.19 mm after outlier removal when measuring sorghum stem diameters with a camera and Time-of-Flight (ToF) system) [10]. Zhou et al. (2024) obtained an RMSE of 1.07 mm for maize stems with approximately 30 mm diameter with a stereoscopic camera system [11]. Mano and Igawa (2017) reached an RMSE of 0.9 cm for rice stems up to 10 cm using a reference scale bar close to the stems and camera system [12]. These studies illustrate that while camera-based techniques offer promising non-contact alternatives, their current accuracy and detection limits can not yet compete with those of conventional manual instruments for many phenotyping applications.

In this paper, an Optical Caliper (OC) for contactless stem diameter measurement is developed. Operating on the principle of shadow formation, the device acquires images of the stem’s shadow profile to determine its diameter. Addressing the need for an affordable, small, portable, and lightweight instrument suitable for non-invasive plant applications, the OC incorporates standard 25 mm optical components. This design provides sufficient space between the optical elements to allow the stem to be positioned within the measurement region without physical contact. The system is developed with the goal of achieving measurement precision comparable to the DC. We demonstrate that the OC achieves measurement precision comparable to that of the DC under stationary conditions on rigid cylindrical standards while surpassing DC performance in field trials on irregular tomato and cucumber stems. This underscores the non-contact OC’s key advantages for real-world biological phenotyping: by eliminating mechanical deformation and averaging over-extended stem segments, it substantially mitigates variability arising from non-circular cross-sections, surface irregularities, and apical tissue compliance, thereby enhancing repeatability and suitability for precision agriculture.

## 2. Materials and Methods

**Figure 1:**
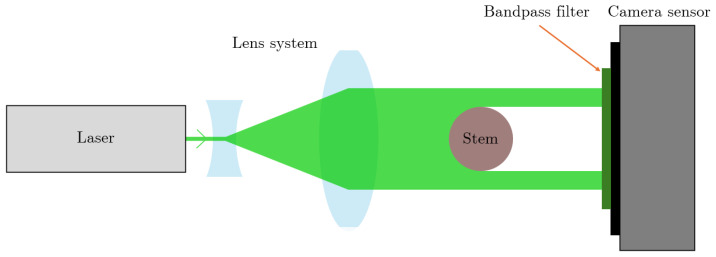
Optical Caliper schematic design. A laser (light grey), together with two lenses (light blue), create a collimated light beam (green). The plant stem (brown) is placed in front of the camera sensor (dark grey and black) which translates the shadow cast by the plant stem to a diameter. A bandpass filter (dark green) is placed in front of the camera sensor to minimize disturbances from external light.

In contrast to conventional calipers, which assess plant stem diameters (or head thickness) via direct physical contact by manually clamping opposing jaws around the stem, the OC enables contactless measurement. Its principle involves projecting a collimated light beam onto the stem to cast a shadow, which is captured by an image sensor for diameter determination. The beam originates from a diode-pumped solid-state laser producing a near-Gaussian round profile, expanded via a telescope configuration. The setup is depicted in Figure 1. The device accommodates measurements near the plant apex (head thickness) or base (stem diameter).

**Figure 2:**
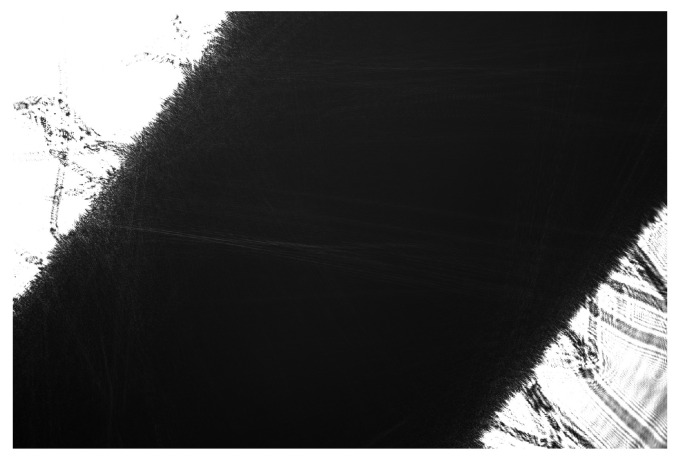
Shadow of a tomato plant head as captured by the camera sensor.

Captured images, as shown in Figure 2, allow for direct estimation of the stem diameter by measuring the pixel distance between the shadow edges, provided the pixel size is known.

Due to the use of a collimated light beam, the stem’s position along the optical axis between the convex lens and the camera sensor has a negligible impact on measurement result. The dimensions of the expanded laser beam and the sensor should be sufficient to accommodate the desired head thickness, which is approximately 10 mm for the head of tomato plants.

### 2.1. Prototype Design

**Figure 3:**
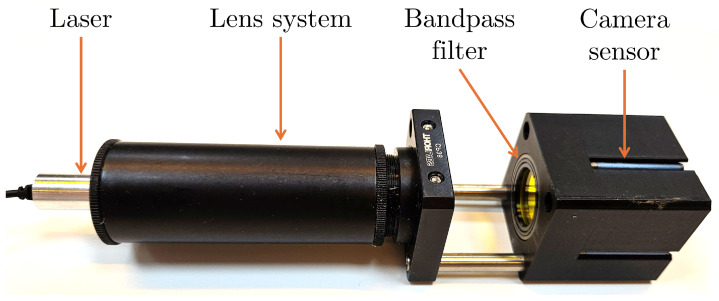
Optical caliper prototype featuring a diode-pumped solid state (DPSS) laser, a lens system for beam expansion and collimation, a bandpass filter for minimizing disturbances from external light sources and a camera sensor for capturing images.

The OC prototype, as shown in Figure 3, employs a MER2-2000-19U3M-L camera from Daheng Imaging, Beijing, China [13], purchased in The Netherlands. This sensor offers a diagonal size of 15.86 mm, a resolution of 5496 × 3672 pixels, and a square pixel pitch of 2.4 µm. To obtain a direct one-to-one correspondence between the projected shadow and the actual plant stem diameter, the camera is operated without a lens. To maximize the measurable range of stem diameters, the camera is positioned such that its sensor diagonal is perpendicular to the projected stem shadow. This orientation leverages the sensor’s aspect ratio to extend the effective measurement span along the diagonal axis. For the aspect ratio of the MER2-2000-19U3M-L camera sensor, this is achieved when the camera is oriented such that the angle between the short axis of the camera sensor and the stem equals 34°.

A continuous-wave 532 nm diode-pumped solid-state laser is employed to generate the collimated light beam. The laser emits a circular beam with a diameter of 3.5 mm at about 0.9 mW. Beam expansion is achieved using a lens system consisting of a bi-concave lens with a focal length of −9 mm positioned directly behind the laser source, followed by a bi-convex lens with a focal length of 35 mm at 26 mm distance. This lens configuration produces a collimated beam with a diameter of approximately 14 mm, sufficient to illuminate the entire area of the camera sensor. The laser irradiance in this configuration is <10 W/m^2^, which is much lower than the irradiance from the sun (up to 1 kW/m^2^) and is considered not harmful to the plant. The laser and lens system are housed within a 25.4 mm diameter lens tube, enabling precise and stable optical alignment. To reduce influence from ambient light, a 532 nm bandpass filter is mounted in front of the camera sensor. The camera is mounted in a custom 3D-printed housing and attached to the lens tube system using two metal rods with a 20 mm gap between the tube and the camera housing, which defines the measurement area. An overview of all the components used in the prototype is shown in Table 1.

The images are captured on a Windows-based laptop with the software Daheng Galaxy Viewer supplied by the camera manufacturer Daheng Imaging. The processing of these images is done immediately after image capture on the same laptop. The total component cost of the OC is €944, excluding the laptop.

### 2.2. Data Processing

**Figure 4:**
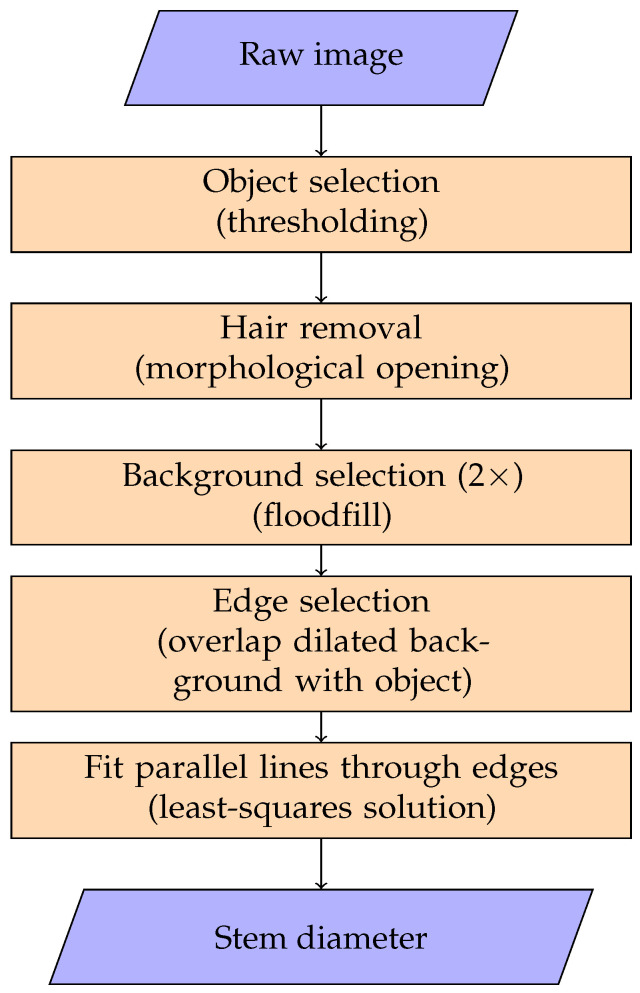
Process of stem diameter estimation from acquired images, implemented in OpenCV [14].

The camera sensor exposure time is chosen such that it is saturated in the area where the light reaches the sensor directly. This creates a sharp contrast in the image of the stem shadow. The distance between the edges of this shadow is then estimated by a series of image-processing steps with OpenCV [14], presented schematically in Figure 4.

The first step is to select the stem object from the acquired gray-value image (5496 × 3672 pixels) using image thresholding. Since the sensor is completely saturated in the background, the foreground pixels can be selected using a threshold value slightly below the maximal pixel intensity, resulting in a binary image.

**Figure 5:**
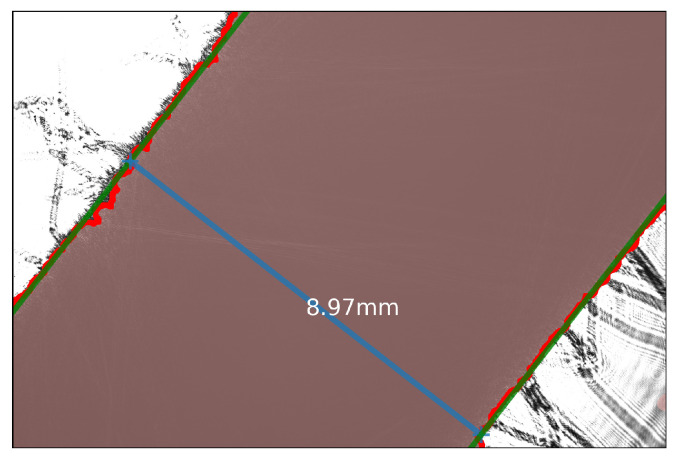
Stem diameter measurement of the projected shadow of a tomato plant head. Colors indicate the steps of the processing pipeline shown in Figure 4: the object after hair removal (brown), the determined edge pixels (bright red), the parallel lines fitted through the edges (green), and an orthogonal line that equals the stem diameter (blue).

The hairs on the stem are irrelevant for the diameter estimation and, consequently, removed in the second processing step with a morphological opening operation on the binary image using a kernel size of 50 × 50. A 50 × 50 size was selected because it provided the best balance between hair removal and preservation of stem edges across the range of stem thicknesses encountered. The resulting binary images represent the estimated stem pixels that are shown in brown in Figure 5.

Subsequently, the two background regions (the white triangles in the upper left and lower right corners in Figure 5) are determined by applying floodfill operations on the (inverted) binary image from multiple seed points along the image border. For each background region, the edge pixels of the stem are determined as the overlap between the background region and the stem pixels dilated using a 5 × 5 kernel. These pixels of both edges are indicated as bright red in Figure 5.

Finally, parallel lines are fitted through the pixels of each edge by estimating their slope (*a*), and intercepts with the axis ($b_{1},b_{2}$) using the least-squares solution. The resulting lines are shown in dark green in Figure 5. The distance between these lines, shown in blue, is considered the stem diameter and is determined by taking into account the pixel size of (2.4 µm) of the camera sensor.

Image processing takes approximately 5 s per image on a standard Windows-based laptop and can be performed immediately after capture. Since 5 min measurement intervals are common practice in greenhouse applications, the processing time is well within operational requirements.

### 2.3. Data Collection

All measurements with the OC were performed by capturing images using the Daheng Galaxy Viewer software version 2.0 provided by the camera sensor manufacturer with the camera’s exposure time set at 1500 µs. The images were then processed to measure the head thickness using the process described in Section 2.2.

The accuracy of the OC was assessed in a laboratory by measuring the diameters of five precision-machined stainless-steel reference cylinders. These cylinders were selected to cover a dimensional range of approximately 4 mm to 12 mm. Each cylinder was measured 30 times. For comparison, the diameter of each cylinder was measured with a DC and a Micrometer Screw Gauge (MSG). The DC and MSG can be considered stationary instruments, as their measurement process requires physically contacting and clamping the cylinder, preventing movement during measurement. The DC used was a KWB 090690 (Stuhr, Germany) and the MSG used was a Mitutoyo 293-240-30 (Kawasaki, Japan).To evaluate the performance of the OC under varying operating conditions, three measurement protocols were employed:

1. OC1: the OC remained stationary both during and between measurements for each cylinder.
2. OC2: the OC was removed and repositioned between successive measurements but remained stationary during each individual measurement.
3. OC3: the OC was used in handheld mode, with the device held freely in the operator’s hand throughout the measurement process.

Greenhouse-based field trials were performed to evaluate the accuracy of the OC on tomato and cucumber head thickness measurements. The tomato measurements were conducted at World Horti Center (Naaldwijk, The Netherlands), while cucumber measurements were performed at Reijm & Zn. (Zevenhuizen, The Netherlands). Both measurements were done in a single day. Several crops were selected, and the head thickness was measured approximately 25 cm below the apex, following the grower’s standard protocol. For the tomato crop, the head thickness of six different plants was measured ten times each. For the cucumber crop, the head thickness of five different plants was measured ten times each. All measurements were taken at a height of 4 to 5 m above the ground using both the DC as the reference method and the OC in handheld mode (OC3). Measurements were conducted under predominantly cloudy conditions, with occasional periods of direct solar illumination. To prevent potential sensor saturation or overexposure caused by high solar irradiance, image acquisition angles were selected to avoid direct sunlight falling onto the camera sensor of the OC.

The mean and standard deviation were calculated for each measurement set, with the reported measurement uncertainty being two times the standard deviation (2σ), which provides a coverage factor (k=2) for a confidence interval of approximately 95%.

## 3. Results and Discussion

### 3.1. Accuracy Assessment Using Reference Cylinders

The accuracy of the OC was benchmarked by measuring the diameters of the five reference stainless-steel cylinders using the OC, DC, and MSG. Table 2 presents the comparative measurement results for the cylinders of varying diameters.

Comparing the mean values, the OC demonstrates good agreement with the established measurement instruments, particularly under stationary operating conditions (OC1 and OC2). For Cylinder 1 (≈4 mm) and Cylinder 2 (≈6 mm), the mean values obtained with the OC (OC1: 3.94 mm and 5.94 mm; OC2: 3.93 mm and 5.92 mm) are in close proximity to those from the DC (3.98 mm and 5.97 mm) and MSG (3.992 mm and 5.986 mm).

It is observed that the mean values obtained with the OC are slightly lower than those from the reference instruments, on average the OC1 are 1.0% smaller, with a maximum deviation of 1.7% for cylinder 5. This systematic underestimation may stem from the current method’s reliance solely on the nominal pixel size provided by the camera sensor manufacturer. Additionally, both imperfect collimation of the light source and diffraction effects can introduce optical distortions that affect edge detection accuracy, particularly for larger diameters. These phenomena may lead to the underestimation of object boundaries in the captured images, thereby contributing to the observed discrepancies between the OC and the MSG measurements. When calibrating the system using the MSG results as a reference, a calibration factor of 1.0093 may be applied to correct the OC results. In that case the maximum deviation of the OC1 results from the reference value provided by MSG reduces to 0.7%. Since measurement repeatability and relative change in stem size are more important for practical applications than absolute dimensional measurements, the absolute calibration of the system is not further taken into account here.

The standard deviation, and consequently the estimated measurement uncertainty, reveal clear differences in the repeatability of the instruments under varying conditions. As expected, the MSG exhibits exceptionally low standard deviations, reflecting its high precision and repeatability. The DC also demonstrates high repeatability, with standard deviations consistently around ±0.01 mm.

Under stationary conditions (OC1), the OC achieves similar repeatability as the DC, with standard deviations of ±0.01 mm. This highlights the capability of the OC to deliver high repeatability when operated under stationary conditions. When the OC is repositioned between measurements (OC2), the standard deviation increases moderately, ranging from ±0.04 mm to ±0.11 mm, indicating that repositioning introduces additional variability, though the results remain reasonably stable.

Handheld operation of the OC (OC3) results in an increase in measurement uncertainty, ranging from ±0.11 mm to ±0.26 mm, depending on cylinder size. This increased variability is likely attributable to minor movements of the device during measurement, as the OC employs a rolling shutter camera, which is sensitive to motion and can introduce artifacts such as geometric distortion or blurring in the captured images. A reduction in the exposure time while increasing the laser power may be a route to reduce sensitivity to instrument movement. Alternatively, the OC may be fixed temporarily to reduce movement, e.g., by mounting it on a robot arm.

### 3.2. Measurement of Crop Head Thickness

To validate the OC’s accuracy on crops, greenhouse trials were performed to measure the head thickness of tomato and cucumber plants. Table 3 presents measurements of the diameter of six individual tomato stems obtained using the DC and the OC prototype in handheld mode.

A notable observation when comparing these results to the measurements on the stainless-steel cylinders (Table 2) is the generally higher measurement uncertainty associated with the DC when used on tomato stems, ranging from ±0.10 mm to ±0.37 mm. This variability is likely due to to the inherent characteristics of biological samples and the nature of contact measurement. Tomato stems are typically not perfectly circular, vary in diameter along their length, and are not rigid, especially in apical sections. Measurements with a DC require physical contact and applied pressure. On a non-rigid and irregular stem, slight variations in the position along the stem, the angle of application, and the amount of pressure exerted by the caliper jaws can lead to minor deformation of the stem. This results in different readings and contributes significantly to the observed increase in standard deviation. The OC’s mean values show reasonable agreement with the DC means for each stem. The estimated measurement uncertainty for the OC (ranging from ±0.14 mm to ±0.31 mm) is often comparable to, or in some cases lower than, the uncertainty observed with the DC on the same samples.

Table 4 presents comparative measurements of the head thickness of five cucumber stems, using the DC and the OC prototype in handheld mode.

The absolute values between DC and OC differs notably. Possibly this is caused by a slight difference in positioning of the DC and OC in combination with stem diameter variability along the stem and the irregular shape of the stem cross section. However, for growers measurement repeatability is more important than absolute dimensions. Consistent with the challenges of measuring irregular biological samples, both the DC and the OC show larger measurement uncertainty when applied to cucumber stems compared to the reference steel cylinders (Table 2). The increased variability is primarily attributed to the inherent square-like geometry of the cucumber stem cross-section. Since both measurement instruments report a single head thickness, the reported value is highly sensitive to the angular orientation of the measurement plane relative to the stem’s flats and corners. The OC uncertainties (ranging from ±0.08 mm to ±0.29 mm) are lower than those recorded by the DC on the same samples, which range from ±0.16 mm to ±0.72 mm. Similar to the findings for tomato stems, the physical pressure required by the caliper jaws likely causes deformation of the soft tissue. The larger uncertainty of the DC for cucumber compared to tomato head thickness is probably caused by the fact that the cucumber stem is significantly softer than the tomato stem. It should also be noted that the OC captures a larger measurement area—especially for smaller diameters—compared to the limited contact points of the DC. By averaging over a greater segment of the stem, the OC reduces the influence of local anomalies and improves repeatability, offering a more robust solution for measuring biologically variable structures.

## 4. Conclusions

In this study we have shown that the optical caliper is a promising tool for plant phenotyping and precision agriculture by evaluating a prototype instrument designed for non-contact measurement of plant stem diameter and head thickness. The OC’s performance was compared with conventional contact measurement instruments (DC and MSG) using both rigid stainless-steel reference cylinders and non-rigid biological samples (tomato and cucumber stems). The measurement results on the stainless-steel cylinders demonstrate that the OC prototype achieves a precision of 0.01 mm, which is comparable to conventional instruments when used under stationary conditions. This slightly increases when the instrument is repositioned between measurements. However, its performance shows some sensitivity to movement when operated as a handheld device, emphasizing the importance of stable mounting when high-precision measurements are desired.

From the measurements on cucumber and tomato stems a comparable or lower standard deviation has been observed for the OC compared to the DC. This is attributed to the non-contact nature of the OC measurement method avoiding deformation of the soft stem tissue. The uncertainty on soft tissue ranges from 0.14 mm to 0.31 mm for tomato and 0.08 mm to 0.29 mm for cucumber, which matches the needs of growers for monitoring head thickness. From the findings we conclude that the developed optical caliper is a viability tool for contactless plant stem measurement, especially in contexts requiring non-invasive monitoring of soft materials.

The high precision observed in stationary measurements is particularly promising for continuous monitoring of stem diameter variation throughout the day, which is also a critical plant parameter for growers. Such temporal tracking could provide valuable insights into plant growth dynamics. In particular, the potential of measuring stem diameter variations at the bottom of a plant with a sub 10 µm resolution is of interest because stem variations at that level can be associated with sap flow. This may turn the OC into a tool for sap flow monitoring, which we are currently further investigating. To underpin the measurement accuracy in this configuration, a more extensive investigation of instrument measurement uncertainties like pixel variation, optical distortion, sensor drift etc., will be needed, since the measurement uncertainty will no longer be dominated by statistical variations as is the case in the handheld configuration.

Future work will focus on the optimal balance between the desired measurement range (maximum stem thickness), spatial resolution, ease of operation and overall system cost. Although a broader measurement range can be achieved to measure thicker stems (e.g., 25 mm), this change will increase the complexity and costs of the system as it requires extra lenses. A significant step towards broader applicability is the development of a standalone, portable version of the OC. Integrating the image acquisition and processing capabilities onto a compact, embedded platform such as a single-board computer (e.g., Raspberry Pi) will eliminate the need for a connected laptop, enabling easier deployment in greenhouses.

## Figures and Tables

**Table 1:** Optical Caliper prototype component list. Daheng Imaging is located in Beijing, China. Thorlabs Inc. is located in Newton, NJ, USA.

Component	Manufacturer	Part Number
Camera sensor	Daheng Imaging	MER2-2000-19U3M-L
Camera housing	N/A	N/A (3D-printed)
Bandpass filter	Thorlabs Inc.	FLH532-10
Laser	Thorlabs Inc.	CPS532-C2
Bi-concave lens	Thorlabs Inc.	LD2568-A
Bi-convex lens	Thorlabs Inc.	LB1811-A
Adjustable lens tube	Thorlabs Inc.	SM1V05
SM1 lens tube	Thorlabs Inc.	SM1M30
Lens adapter	Thorlabs Inc.	AD9T
Cage plate	Thorlabs Inc.	CP36
Cage rod	Thorlabs Inc.	ER2

**Table 2:** Comparison of measurement results for stainless-steel cylinders with varying diameters using different instruments and operating conditions. OC1: Optical Caliper under fixed, stationary conditions; OC2: Optical Caliper stationary during measurement but repositioned between measurements; OC3: Optical Caliper used in handheld mode. Each cylinder was measured 30 times using all instruments and protocols, and the measurement uncertainty was estimated as twice the standard deviation (2σ) of the repeated measurements.

Cylinder	MSG [mm] ± 0.001	DC [mm] ± 0.01	OC1 [mm] ± 0.01	OC2 [mm]	OC3 [mm]
1	3.992	3.98	3.94	3.93 ± 0.05	3.91 ± 0.11
2	5.986	5.97	5.94	5.92 ± 0.04	5.88 ± 0.14
3	7.991	7.98	7.97	7.95 ± 0.12	7.90 ± 0.23
4	9.990	9.98	9.93	9.91 ± 0.09	9.81 ± 0.26
5	11.989	11.98	11.79	11.77 ± 0.11	11.78 ± 0.17

**Table 3:** Tomato head thickness measurements with a Digital Caliper and Optical Caliper in handheld mode. Each stem was measured 10 times with both calipers. The measurement uncertainty was estimated as twice the standard deviation (2σ).

Stem	Digital Caliper [mm]	Optical Caliper [mm]
1	11.36 ± 0.37	11.11 ± 0.31
2	9.82 ± 0.26	9.66 ± 0.15
3	10.07 ± 0.10	10.47 ± 0.16
4	11.00 ± 0.12	10.80 ± 0.14
5	9.13 ± 0.22	9.08 ± 0.25
6	8.06 ± 0.17	8.21 ± 0.15

**Table 4:** Cucumber head thickness measurements with a Digital Caliper and the Optical Caliper in handheld mode. Each stem was measured 10 times with both calipers. The measurement uncertainty is reported as twice the standard deviation (2σ).

Stem	Digital Caliper [mm]	Optical Caliper [mm]
1	9.13 ± 0.51	8.61 ± 0.29
2	8.50 ± 0.72	8.28 ± 0.13
3	8.52 ± 0.16	8.66 ± 0.08
4	9.86 ± 0.30	8.64 ± 0.19
5	6.80 ± 0.34	6.29 ± 0.23

## Data Availability

The data gathered during this study is openly available via https://hhs.data.surf.nl/s/nqnFYBf42KPA75P (accessed on 10 February 2026).

## Abbreviations

The following abbreviations are used in this manuscript:

OC	Optical Caliper
DC	Digital Caliper
MSG	Micrometer Screw Gauge
